# Combining LC–MS/MS and hollow-fiber infection model for real-time quantitation of ampicillin to antimicrobial resistance

**DOI:** 10.4155/fsoa-2018-0055

**Published:** 2018-10-17

**Authors:** Adarsh Gandhi, Murali Matta, Tesfalem Zere, James Weaver

**Affiliations:** 1 Division of Applied Regulatory Science, Office of Clinical Pharmacology, Food & Drug Administration, 10903 New Hampshire Avenue, Silver Spring, MD 20993, USA

**Keywords:** ampicillin, antibiotic resistance, hollow-fiber, Luria–Bertani broth, mass spectrometry

## Abstract

Although a marked decrease in mortality associated with bacterial infections is attributed to the discovery of antibiotics, antibiotic resistance has become a global health concern due to their misuse. A dynamic *in vitro* hollow-fiber system was used to study antibiotic resistance in *Escherichia coli* using ampicillin. An LC–MS/MS assay was validated for quantitative analysis of ampicillin in Luria–Bertani broth. The assay was linear from 0.10–50.00 μg/ml. The assay met acceptance criteria for inter- and intra-assay precisions and accuracies across three quality controls. Stability of ampicillin was confirmed at three different storage conditions. *In vitro* data were similar to simulated plasma PK data further confirming the appropriateness of the experimental design to quantify antibiotics and study occurrence of antimicrobial resistance in real-time.

## Lay abstract

Urinary tract infections are becoming untreatable with the rise of antibiotic resistance. *Escherichia coli* is the cause of most urinary tract infections, one of the most common infections that require treatment with antibiotics. The utility of a hollow-fiber system, which is a dynamic system mimicking human disposition of antibiotics more closely than static systems, can help design single or combination drug dosing regimens for antibiotics based on their pharmacokinetic profiles. The data generated from these studies can be utilized to help understand the time when bacteria start developing resistance and aid in adjusting the drug combinations and their doses.

The discovery of antibiotics in the 1920s led to optimism that infections can be controlled and prevented. However, antimicrobial resistance poses a growing threat to public health and the provision of healthcare, especially in the developing world. In addition to the newly discovered diseases, reemergence of diseases once controlled, and, more specifically, rapid emergence of multidrug-resistant pathogens (superbugs), constitute a major public health threat, making them among the top 20 causes of death. The Centers for Disease Control and Prevention (CDC) states that the increasing impact of antibiotic resistance-related infections accounts for an estimated 23,000 deaths and more than 2 million illnesses each year in the USA alone [1].

Urinary tract infections (UTIs) are common in both outpatient and inpatient settings. Out of the various life-threatening diseases such as tuberculosis, pneumonia, respiratory tract infections, and so forth, UTI is one of the major nosocomial infections, affecting one in every three women by the age of 24 [2,3]. Uropathogenic *Escherichia coli* is the predominant pathogen in uncomplicated UTIs followed by *Klebsiella pneumoniae and Staphylococcus saprophyticus.* For complicated UTIs, *E. coli* is also the predominant pathogen followed by followed by *Enterococcus species* and *Klebsiella pneumoniae.* [4]. *Proteus mirabilis* is also found in UTIs. Ampicillin (AMPI; Figure 1) is a β-lactam antibiotic, with a minimum inhibitory concentration (MIC) of 4 μg/ml against sensitive strains of *E. coli* (CFT-073) and demonstrates time-dependent killing, where the duration of time the free concentration is maintained above the (fT > MIC) correlates best with its bacterial killing [5]. The drug is acidic in nature and it acts by inhibiting the third and the final stage of bacterial cell wall synthesis, leading to cell lysis. Although AMPI is one of the most prescribed antibiotics compared with other β-lactams, several reports show a high prevalence in developing resistant *E. coli* strains [6,7]. A recent review discussed the potential as well as shortfalls of combination therapy with AMPI and ceftriaxone for enterococcal endocarditis in humans [8]. After performing a systematic review of the clinical studies, the authors concluded that ampicillin and ceftriaxone may be considered in cases where patients are at high risk of nephrotoxicity or when synergy is required in the treatment of high-level aminoglycoside resistance organisms. Thus, although effective against endocarditis, limited data warrant further randomized, noninferiority trials to definitively address the efficacy and safety of this combination therapy.

**Figure 1.**
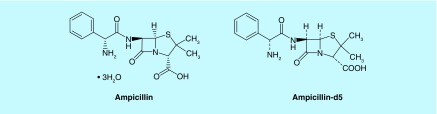
**Chemical structures of ampicillin and ampicillin-d5 (IS).**

To quantify the emergence of antibiotic resistance we utilized the *in vitro* hollow-fiber infection model (HFIM) [9]. Several advantages of using the HFIM include: confined bacterial exposure, strong *in vitro* to *in vivo* extrapolations to mimic concentrations observed in *in vivo* infections, long experimental durations (days or weeks) and a dynamic two compartment system allowing rapid equilibration of the drug [10]. Timely sample collection can easily be performed without significantly affecting the bacterial population along with minimizing the risk of accidental exposure to drug-resistant and highly pathogenic organisms. Large numbers of organisms can be tested in one experiment so the emergence of drug resistance is easily quantified. Another strength of HFIM is that it can mimic any pharmacokinetic profile allowing precise and controlled antimicrobial testing.

In studying the kinetics of the emergence of antibiotic resistance it is critical to determine accurately the actual amount of drug present in the HFIM system. Liquid chromatography–tandem mass spectrometry (LC–MS/MS)-based quantitative analysis of AMPI in human plasma, urine or tissue is documented in the literature. [11,12]. However, here we present a novel method for accurate quantitation of AMPI in Luria–Bertani (LB) broth in support of antimicrobial resistance study using the *in vitro* hollow-fiber infection model.

## Materials & methods

### Chemicals & reagents

Ampicillin hydrochloride (purity > 95%) was purchased from Sigma-Aldrich (MO, USA). The internal standard (IS; ampicillin d-5 [AMPI-d5], purity > 98.8%) was purchased from Toronto Research Chemicals (CA, USA). Ammonium acetate for buffer preparation was purchased from Sigma-Aldrich. Formic acid, LC–MS grade methanol, acetonitrile and water were purchased from Fisher Scientific (NH, USA).

### Preparation of stock solutions, calibration standards & quality controls in Luria–Bertani broth

Primary stock solution (1.0 mg/ml) of AMPI was prepared in methanol. These stock solutions were stored at -20°C. From these stock solutions, appropriate dilutions were made in 60% methanol to get working spiking solutions. These spiking solutions were used for spiking in blank Luria-Bertani broth, to make calibrants and QC samples. The matrix used to prepare calibrants and QC samples, was screened for potential interference at the retention times and mass transitions of AMPI and its IS (AMPI-d5), respectively. The matrix was free of significant interference. In addition to blank and zero calibrants, a set of nine nonzero calibrants (0.10–50.00 μg/ml) and five levels of QC (0.10, 0.30, 8.00, 40.00 and 50.00 μg/ml) were prepared with drug-freeLBbroth. All the calibrators and QCs were stored at -20°C until ready for extraction.

Primary stock solutions of IS (AMPI-d5) were prepared at 1.0 mg/ml and stored at -20°C. The stock solutions were diluted in acetonitrile for preparing extraction solvent containing 10.00 μg/ml of AMPI-d5 and stored at -20°C.

### *In vitro* hollow-fiber infection model

The HFIM system has been extensively described elsewhere [13,14]. *E. coli* strain CFT073, obtained from ATCC, was used in this study. Frozen *E. coli* stock cultures were inoculated in LB broth (Luria-Bertani) and were incubated at 37°C overnight. The overnight cultures were subcultured (1:1000) into fresh LB broth, incubated at 37°C and were grown until they reach mid-exponential growth phase (OD_600_ = ∼ 0.5, ∼ 10^∧^8 CFU/ml). 20 ml of the mid-exponential phase *E. coli* culture was then inoculated into the hollow fiber. The bacteria were exposed to humanized pharmacokinetic concentrations of ampicillin for 10 days. Human pharmacokinetic concentrations of ampicillin were produced by computer control of the flow rates of the drug and diluent flowing through the inner lumens of the hollow fibers. The dosing regimen for ampicillin was set up to model a thrice daily dosing [15]. Bacterial and drug samples were collected at different time points for further analysis.

### Sample preparation

Protein precipitation method was employed for extraction of AMPI from *in vitro* LB broth samples. Briefly, 50 μl of standard, QC or study samples were extracted with 100 μl of IS-spiked acetonitrile in FiltrEX™ 96-well filter plates with hydrophilic 0.2 μ PVDF Membrane (Corning Inc., NY, USA). The samples were centrifuged at 4000 rpm for 5 min at 8°C.

### LC–MS/MS instrumentation & analytical conditions

A 1290 Agilent Infinity (Agilent Technologies, CA, USA) UHPLC system and an auto sampler were used as the liquid chromatograph. 3 μl aliquot of the processed sample was chromatographed on a Kinetex HILIC column (2.1 × 50 mm, 2.6 μl; Phenomenex, CA, USA). The column oven temperature was maintained at 25 ± 5°C. The optimized mobile phase was composed of 2 mM ammonium acetate buffer (pH 4.76) and acetonitrile. For resolving AMPI, 0.2% formic acid was added to acetonitrile. The flow rate was set at 0.3 ml/min. Data acquisition was achieved using a Waters Quattro Micro API Tandem quadrupole mass spectrometer (Waters Corporation, MA, USA) equipped with an ESI source maintained at 120°C. Specifically, the mass spectrometer was operated in positive ion mode for 3.0 min. Cone voltage was set at 21. Desolvation temperature was set at 350°C. Detection of the ions was carried out in multiple-reaction monitoring mode (MRM), by monitoring the transition pairs of *m/z* 349.9 → 105.9 and 354.9 →110.9 for AMPI and AMPI-d5, respectively. Analytical data were acquired and processed using Mass Lynx™ quantitation software.

### Method validation

Validation parameters like selectivity, sensitivity, specificity, precision and accuracy, recovery, matrix effect, linearity, dilution integrity, stability and injection carryover parameters were successfully validated in this assay as per the US FDA Guidance for Bioanalytical Method Validation (FDA, 2013). Specificity of the method was designed to investigate interference from six blank LB broth samples. The response of background noise at the retention time of the analytes was acceptable if it was less than 20% of the response of lower limit of quantification (LLOQ) and less than 5% of the response of working concentration of ISs. Selectivity was assessed by comparing the peak area responses between AMPI spiked LB broth and extracted with blank acetonitrile, or blank LB broth extracted with IS spiked acetonitrile. Sensitivity was determined by analyzing six replicates of LB broth spiked with corresponding LLOQ. The six replicates should have a precision of ≤20% and an accuracy of ± 20%. Calibration curve was constructed in the range of 0.10–50.00 μg/ml. Linearity of the plot was evaluated based on slope, intercept and correlation coefficient values derived from least square regression analysis. The correlation coefficient (r^2^) was more than 0.99 in three different analytical runs. Matrix effect was investigated to check that precision, selectivity and sensitivity are not compromised by the biological matrix. Matrix factor was assessed with blank LB broth. For evaluation of intraday precision and accuracy, six replicate QCs were analyzed with four different concentration levels: LLOQ; LQC (low-quality control); MQC (medium-quality control); and HQC (high-quality control), on the same day. For evaluation of inter-day precision and accuracy, six replicate QCs were analyzed from three different runs on three different days. The acceptance criteria for intraday and interday accuracy were set at ± 15% deviation (SD) from the nominal value, except LLOQ, where it must be ± 20%. For precision, the coefficient of variation (%CV) should be ± 15% for all the QCs, except for the LLOQ, where it should be ± 20%.

The recovery was evaluated by comparing the peak areas of analytes in spiked QCs (six each of LQC, MQC and HQC) with those of analytes in post-extracted samples. The recovery of IS were determined in a similar way at working concentration of 10.00 μg/ml for AMPI-d5.

Stability studies were conducted to evaluate the stability of analytes under different storage conditions. Auto sampler storage stability at 4°C (up to 72 h), freeze–thaw stability at -20°C (three cycles) and short-term stability at room temperature (up to 12 h) experiments were performed at LQC and HQC for AMPI. The measured concentrations were compared with the nominal values. Samples were stable if the deviation was ± 15% from nominal values.

Injection carryover tests were conducted to assess carryover (if any) from the upper limit of quantification (ULOQ) sample. The acceptance criterion was peak area response in the blank sample injected immediately after ULOQ should be less than 20% of the LLOQ response.

### Data analysis

For all data analyses, sample concentrations were obtained using data from the calibration curve prepared within the batch using a linear regression with peak area ratio (drug/IS area responses) against concentration (x), with 1/x^2^ weighting as the mathematical basis of the quantification.

## Results & discussion

### Mass spectrometry optimization

A LC–MS/MS method was developed with the purpose to have a sensitive and selective method suitable for quantification of AMPI from *in vitro* studies. An analytical method should be efficient to quantify the drug candidates in presence of matrix components effectively. Hence, we chose a LC–MS/MS technique due to its inherent selectivity and sensitivity.

The MS parameters of AMPI were optimized in positive ionization mode. A 1 μg/ml tuning solution of AMPI along with the IS was infused to optimize the mass spectrometry conditions. Precursor ion signals were increased by suitably altering the capillary spray voltage and the final values were set at 4V in Q1 MS. The most intense and consistent product ion in Q3 MS was obtained by optimizing the collision energy. Finally, the source parameters such as desolvation temperature (350°C), cone voltage (21V), desolvation gas (500 psi) and cone gas (25 psi) were optimized to obtain an adequate and reproducible response. The mass spectra revealed protonated molecular ions by monitoring the precursor→product ion mass transitions as follows: *m/z* 349.9→105.9 for AMPI; *m/z* 354.9→110.9 for AMPI-d5.

### Liquid chromatography & selectivity

**Figure 2.**
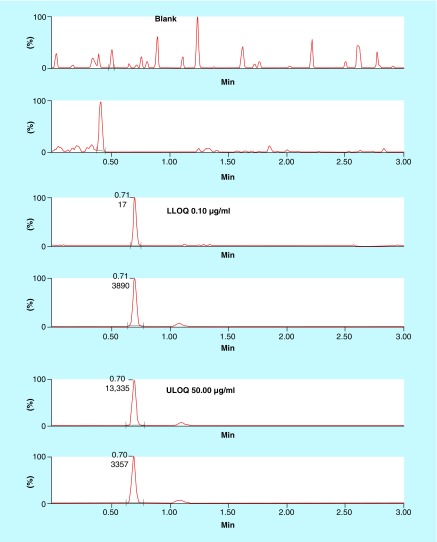
**Representative chromatograms of ampicillin and ampicillin-d5 in blank Luria–Bertani broth, lower limit of quantification sample and upper limit of quantification sample.** LLOQ: Lower limit of quantification; ULOQ: Upper limit of quantification.

Chromatography conditions such as buffer strength, analytical column, and mobile composition and its flow rate were suitably altered to achieve better separation of the analytes from matrix components. Organic modifiers such as acetonitrile and methanol in combination with acidic modifiers such as formic acid were evaluated critically for their suitability to achieve better chromatographical resolution. Use of 2 mM ammonium acetate (pH 4.76) as the mobile phase enhanced the response and improved the reproducibility. Finally, 2 mM ammonium acetate:acetonitrile with 0.2% formic acid in gradient condition was chosen as the choice of mobile phase combination to elute AMPI and AMPI-d5. Acetonitrile with 0.2% formic acid was chosen instead of 100% acetonitrile for better peak shapes and sensitivity. Upon injection of 3 μl sample on a Kinetex HILIC column (2.1 × 50 mm, 2.6 μm) with a flow rate of 0.3 ml/min at controlled room temperature, AMPI/AMPI-d5 eluted at 0.69 min in a total run time of 3.0 min. Representative chromatograms of AMPI/AMPI-d5 in blank, LLOQ and ULOQ samples are shown in Figure 2. It should be noted that the proportion of aqueous to organic solvent in the final sample and mobile phase solution on column were observed to be very crucial for sharp peak shapes and consistent retention times.

### Sample preparation

Selection of a proper extraction method is very important to get good and reproducible recovery with negligible or no matrix effect. In the present study, we adopted a simple sample extraction procedure to extract the analytes from LB broth. The reason for selecting acetonitrile as an extraction solvent was its compatibility with the mobile phase of the proposed method and its higher polarity, which allows it to permeate easily through the filter membranes and enhance the recovery. Moreover, the precision and accuracy results obtained during the entire course of validation results support this extraction methodology. LB broth, a general purpose media, was selected as it has been a widely used growth medium for bacteria.

For bioanalytical methods, use of stable labeled isotope standards of analytes are recommended as ISs to increase method precision and limit the variability in ionization, chromatography and extraction, and to help in tracing potential matrix-related challenges. For AMPI, we used AMPI-d5 as an IS and to normalize against any ion enhancement or suppression resulting from the matrix.

### Linearity

**Figure 3.**
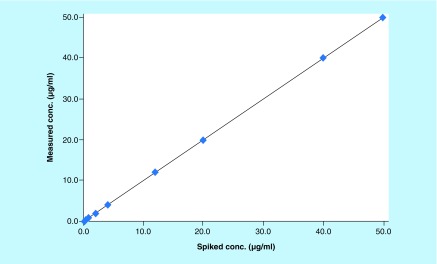
**Representative standard curve of ampicillin in Luria–Bertani broth.**

Linearity of the method was evaluated at nine nonzero concentrations of AMPI (0.10–50.00 μg/ml). After comparing the two weighting models (1/x and 1/x^2^), a regression equation with a weighting factor of 1/x^2^ of AMPI to AMPI-d5 concentration was found to produce the best fit for the concentration–peak area response relationship (Figure 3). The mean correlation coefficient of weighted calibration curves generated during validation was > 0.99 from three independent runs.

### Sensitivity

The LLOQ for AMPI was 0.10 μg/ml in LB broth, at which the mean values were ± 10% of the spiked values and intra- and interday coefficients of variation were < 13% with intra- and interday accuracies were 90 and 91%, respectively.

### Precision & accuracy

Results for intra- and inter-day precision and accuracy for 4 QC levels are illustrated in Table 1. The intra- and inter-day results were found to be within acceptable limits as per the 2013 FDA Guidance on Bioanalytical Method Validation. There was no constant direction to the bias (i.e., + or -) for QC samples and the mean values were ± 15% of the spiked values. Imprecision was acceptable, as indicated by both intra- and inter-day coefficients of variation of <15% at all concentrations of AMPI. Similarly, the intra- and inter-day accuracies were ± 15% of the spiked values.

**Table 1.** **Intra- and inter-batch precision and accuracy for ampicillin.**

**Quality control conc. (μg/ml)**	**LLOQ (0.10)**	**LQC (0.30)**	**MQC (8.00)**	**HQC (40.00)**
**Intra-assay precision and accuracy (n = 6 at each QC level)**				

Mean	0.09	0.32	7.65	38.82

SD	0.01	0.01	0.22	0.37

% CV	11	3	3	1

% Accuracy	90	105	96	97

**Inter-assay precision and accuracy (n = 18 at each QC level)**				

Mean	0.09	0.31	7.49	37.84

SD	0.01	0.02	0.42	1.35

% CV	11	6	6	4

% Accuracy	91	103	94	95

HQC: High quality control; LLOQ: Lower limit of quantification; LQC: Low quality control, MQC: Medium quality control.

### Extraction recovery and matrix effect

In this study, there was significant matrix effect (ion suppression) observed in LB broth for AMPI tested at LQC, MQC and HQC levels. Thus, for recovery calculations, post-extracted LB broth spiked with analytes were chosen to calculate the relative recoveries of AMPI and AMPI-d5. The mean recovery across the different QCs for AMPI was 101, and 100% for AMPI-d5 and was consistent with %CV < 4% across the three QCs (Table 2).

**Table 2.** **Extraction recovery and matrix effect of ampicillin in Luria&Bertani medium. n = 6 at each QC level.**

**AMPI conc. (μg/ml)**	**LQC (0.30)**	**MQC (8.00)**	**HQC (40.00)**
Mean percent extraction recovery	105	100	99

Mean percent extraction recovery (AMPI-d5)	100		

Matrix factor (IS normalized)	1.1	1.0	1.1

AMPI: Ampicillin; High-quality control; LQC: Low-quality control, MQC: Medium-quality control.

For matrix effect, a value of 100% indicates that the responses in the mobile phase and in post-extracted matrix are same and no absolute matrix effect is observed. A value >100% indicates ion enhancement and a value <100% indicates ion suppression. However, matrix effects can be overcome by ISs which correct for ion enhancement/suppression. Matrix effect was assessed by calculating the matrix factor at three QC levels. Matrix factor is calculated as the peak area ratio of analyte with or without IS in extracted LB broth (post spiked) to neat samples (mobile phase). A value between 0.8 and 1.2 for matrix factor denotes no significant matrix effect. Similarly, IS-normalized matrix factor was calculated by dividing the analyte matrix factor by the IS matrix factor. As shown in Table 2, matrix factor normalized with the IS (AMPI-d5) was within the range of 0.8–1.2 with precisions <6% for AMPI. The results indicate that the sample preparation method was specific to AMPI in the presence of IS to rule out any matrix interference.

### Stability studies

Stability of AMPI in postextracted samples stored at 4°C for up to 72 h in auto sampler, subjected to different freeze–thaw cycles and on bench top at room temperature for up to 12 h in LB broth were evaluated. As shown in Table 3, AMPI was stable: in the auto sampler at 4°C for up to 72 h, three freeze–thaw cycles at -20°C mean and at room temperature on the bench top for up to 12 h. Percent accuracies for all the stability tests for AMPI were found to be ± 15% of the spiked concentrations at LQC and HQC levels.

**Table 3.** **Stability data for ampicillin in Luria–Bertani medium. **

**Stability test**	**Spiked QC conc. (μg/ml)**	**Mean ± SD (μg/ml)**	**%CV (precision)**	**% Stability (accuracy)**
Autosampler^†^	0.30	0.31 ± 0.03	10	105

	40.00	37.98 ± 1.54	1	95

Freeze–thaw^‡^	0.30	0.33 ± 0.02	6	109

	40.00	38.72 ± 0.46	1	97

Benchtop^§^	0.30	0.32 ± 0.02	6	108

	40.00	38.54 ± 0.51	1	96

^†^Up to 72 h in autosampler at 4°C.

^‡^Up to 3 freeze–thaw cycles at -20°C.

^§^At room temperature for up to 12 h.

n = 6 at each QC level.

CV: Coefficient variant; QC: Quality control; SD: Standard deviation.

### *In vitro* sample analysis

**Figure 4.**
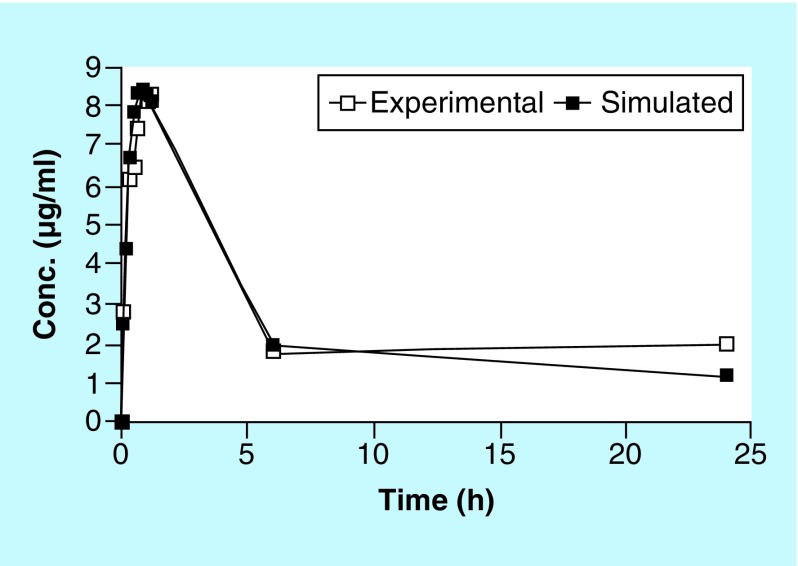
**Correlation of *in vitro* experimental versus simulated PK data for ampicillin in Luria&Bertani broth.**

As demonstrated in Figure 4, the closeness of the *in vitro* experimental data from the hollow-fiber infection model and simulated plasma PK data (using Phoenix WinNonlin v.1.6) up to 24 h, suggests the reliability of the model in measuring antibiotic concentrations in real time. These data also demonstrate very minimal nonspecific adsorption of AMPI to the hollow-fiber capillary tubes. Collectively, these data can help guide antibiotic-resistance studies for making early decisions for predicting the occurrence of resistance.

## Conclusion

For the first time, we have developed and validated a LC–MS/MS method for determination of AMPI to support *in vitro* antibacterial drug resistance studies using a hollow-fiber infection model. An LLOQ of 0.10 μg/ml for AMPI easily meets the requirements for drug detection several folds below the intended MIC. The discussed method is simple, rapid, sensitive and selective for quantification of AMPI and can be applied for *in vitro* evaluation of drug efficacy against different strains of *E. coli*.

## Future perspective

UTIs are the most common bacterial infections adding billions of dollars to healthcare revenues each year. More and more bacteria are becoming increasingly resistant to the only treatment option: antibiotics. Research has been limited in bringing new antibiotics to the clinic and major effort is spent on revamping old antibiotics for curing bacterial infections. Moreover, recurring UTIs suggest that antibiotics are not very effective for all UTIs. Our study on emergence of antimicrobial resistance using a hollow-fiber infection model offers a rational approach in a dynamic environment to study resistant bacterial populations and make appropriate modifications in dosing regimen or implementing combination therapy. The use of probiotics to prevent vaginal urinary tract pathogenic *E. coli* colonization and the use of an immuno-stimulatory uropathogen extract (SolcoUrovac), are currently in clinical trials to determine efficacy in preventing recurrent UTIs. Another preventative and attractive strategy is vaccination, and experimental vaccines have been shown to be effective at blocking host–pathogen interactions, thus preventing the establishment of UTIs, especially in primates. Moving forward, an effective combination of traditional and innovative prevention and treatment strategies will be needed to combat the threat of emerging antibiotic resistance. Our forthcoming manuscripts on the emergence and prevention of resistant colonies using combination (two or three antibiotics) will highlight the role of *in vitro* hollow-fiber infection model in studying bacterial resistance.

**Executive summary**

- Antimicrobial resistance is a social, psychological and economic burden globally.
- Understanding the key mechanisms behind resistance development would be the key for effective management of bacterial infections.
- Our study using a hollow-fiber infection model can be exploited as a promising *in vitro* tool in understanding the course of emerging resistant colonies and the corresponding drug concentrations mimicking human pharmacokinetics.
- Our manuscript is the first ever to measure ampicillin concentrations in Luria-Bertani broth and its application to antimicrobial resistance study.
